# Bioactive Compounds and Sensory Quality in Chips of Native Potato Clones (*Solanum tuberosum* spp. *andigena*) Grown in the High Andean Region of PERU

**DOI:** 10.3390/foods12132511

**Published:** 2023-06-28

**Authors:** Carlos A. Ligarda-Samanez, Henry Palomino-Rincón, David Choque-Quispe, Elibet Moscoso-Moscoso, José C. Arévalo-Quijano, Mary L. Huamán-Carrión, Uriel R. Quispe-Quezada, Jenny C. Muñoz-Saenz, Edgar Gutiérrez-Gómez, Domingo J. Cabel-Moscoso, Reynaldo Sucari-León, Yolanda Aroquipa-Durán, Antonina J. García-Espinoza

**Affiliations:** 1 Food Nanotechnology Research Laboratory, Universidad Nacional José María Arguedas, Andahuaylas 03701, Peru; elibetmm22@gmail.com; 2 Nutraceuticals and Biomaterials Research Group, Universidad Nacional José María Arguedas, Andahuaylas 03701, Peru; dchoque@unajma.edu.pe (D.C.-Q.); huamancarrionmary@gmail.com (M.L.H.-C.); 3 Research Group in the Development of Advanced Materials for Water and Food Treatment, Universidad Nacional José María Arguedas, Andahuaylas 03701, Peru; 4 Agroindustrial Engineering, Universidad Nacional José María Arguedas, Andahuaylas 03701, Peru; 5 Water and Food Treatment Materials Research Laboratory, Universidad Nacional José María Arguedas, Andahuaylas 03701, Peru; 6 Department of Education and Humanities, Universidad Nacional José María Arguedas, Andahuaylas 03701, Peru; jcarevalo@unajma.edu.pe; 7 Agricultural and Forestry Business Engineering, Universidad Nacional Autónoma de Huanta, Ayacucho 05000, Peru; uquispe@unah.edu.pe; 8 Human Medicine Faculty, Universidad Peruana los Andes, Huancayo 12006, Peru; d.jmunoz@upla.edu.pe; 9 Engineering and Management Faculty, Universidad Nacional Autónoma de Huanta, Ayacucho 05000, Peru; egutierrez@unah.edu.pe (E.G.-G.); rsucari@unah.edu.pe (R.S.-L.); 10 Ambiental Engineering, Universidad Nacional San Luis Gonzaga, Ica 11001, Peru; jesus.cabel@unica.edu.pe (D.J.C.-M.); antonina.garcia@unica.edu.pe (A.J.G.-E.); 11 Professional Nursing School, Universidad Nacional Autónoma Altoandina de Tarma, Junín 12731, Peru; yaroquipa@unaat.edu.pe

**Keywords:** native potato clones, bioactive compounds, antioxidant capacity, snacks, chips, organoleptic analysis

## Abstract

Native potatoes (*Solanum tuberosum* spp. *andigena*) have diverse pigments and are cultivated in Peru’s high Andean regions; they are characterized by containing bioactive compounds that prevent various degenerative diseases. The study aimed to evaluate the physicochemical and sensory quality in chips of native potato clones grown at 3496 m altitude, for which the potatoes were cut into slices and fried in extra virgin olive oil at 180 °C for 200 s. This was determined by proximal analysis, reducing sugars, minerals, color, antioxidant capacity (AC), total phenolic compounds (TPC), and anthocyanins in fresh and chips; an instrumental characterization by FTIR and SEM and sensory tests were also performed. The native potatoes presented low moisture and reduced sugar contents; when frying, their bioactive properties improved, increasing AC, TPC, and trace elements, such as K, Mg, Ca, P, Fe, and Zn. To conclude, fresh clones have high yields in the field and are an essential source of nutrients and bioactive; the salt-free chips of clone B presented better physicochemical properties and greater sensory acceptance, closely followed by clone A. Both clones could be used as raw material by food companies that produce snacks to benefit high Andean agricultural producers.

## 1. Introduction

The farmers in the high Andean region of Perú play a crucial role in preserving the genetic diversity of native potato crops. There are different varieties adapted to extreme conditions with unique properties that are undervalued by agribusiness; that is why it is necessary to study processing alternatives to reduce poverty and food insecurity in the country’s poorest areas [1]. Native potato clones contain bioactive compounds that prevent various degenerative diseases due to their high content of polyphenols, anthocyanins, flavonoids, carotenoids, vitamins C, B_3_, and B_6_; they also contain minerals, such as potassium, iron, zinc, phosphorus, and magnesium, as well as various flavors, colors, and shapes, which are valued in the preparation of different foods [2,3,4,5,6,7]. The cultivation and conservation of native pigmented potatoes in Peru are rooted in the ancestral culture of the high Andean populations [8], and the participation of men and women in agricultural work is differentiated [9].

Native potatoes do not contain pesticides, since their cultivation is organic [10]; they also have low moisture, low level of reducing sugars, and lower oil absorption capacity during frying, compared to known commercial varieties, which contributes to a reduction in energy cost by eliminating water, and a low level of reducing sugars reduces the appearance of the Maillard reaction and bitter taste [3,11]. The low water content of potato chips is around 2%, and in the case of lipids, relatively high values between 30 and 39% [12], dry matter percentages between 21 and 25%, starch between 16 and 20%, and reducing sugar contents of less than 0.25% are considered adequate parameters in raw materials for potato chips [13].

In many parts of the world, potato chips occupy a prominent place in the snack market; these products are obtained by frying potato slices in different vegetable oils [14], allowing for a crunchy and delicious product, sensory valued by most people [12,15]. This is also linked to consumers currently preferring healthier and more palatable snacks, so developing potato chips with attractive sensory properties and health benefits is vital [3,16]. Frying can generate unique sensory properties in the final product, but also generates adverse effects, such as high caloric content due to fat absorption and toxic compounds, such as oxysterols and acrylamides [17]. Oil absorption is one of the main drawbacks and is influenced by temperature, time, food characteristics, the formation of wetting agents, and treatments before and after frying [4].

The development of new products is related to the knowledge of consumer preference and acceptance; a valuable tool for this task is so-called sensory evaluation. In particular, preference and acceptance tests of the hedonic scale are used, with which it is possible to know the real use of food in the market and to observe significant differences between products. Non-parametric tests, such as Friedman-Wilcoxon [18], Kruskal-Wallis, and Mann-Whitney [3,19], are used to process sensory evaluation data.

The native potatoes of Peru contain better nutrients than commercial varieties, which could benefit the health of those who eat this food [20]. The problem is that their consumption needs to be diversified, which could lead to their replacement by improved varieties over time, causing this type of potato to disappear [21]. Its low diversification and demand are because very few companies use them in their technological processes, especially fried flakes [4]. Therefore, the present study seeks to add value to these underutilized raw materials, on account of higher field yields, high dry matter content, low levels of reducing sugars, and better nutritional and functional properties than other native potatoes grown in Peru and other countries in the Andean region of South America.

The research aim was to obtain chips of native potato clones to evaluate their physicochemical and sensory qualities.

## 2. Materials and Methods

### 2.1. Materials

Six clones of native potato (*Solanum tuberosum* spp. *andigena*) with pigmented pulps from the district of San Jerónimo, province of Andahuaylas and Apurímac Region—Peru (13°38′43.8″ S, 73°18′22.5″ W, and 3496 m altitude) were evaluated. The samples were kindly provided by the engineer José Palomino Flores of the company “*SEMPAL S.R.L.*” and coded as clone A (blue yellow 507130.1), clone B (Qeqorani 511188.2), clone C (red 303903.602), clone D (pink pulp 21.2021), clone E (purple pulp 511110.5), and clone F (purple pulp Wenqos selection). Samples of 20 kg of each native potato clone were used, and all physical and chemical analyses were done in triplicate.

**Figure 1:**
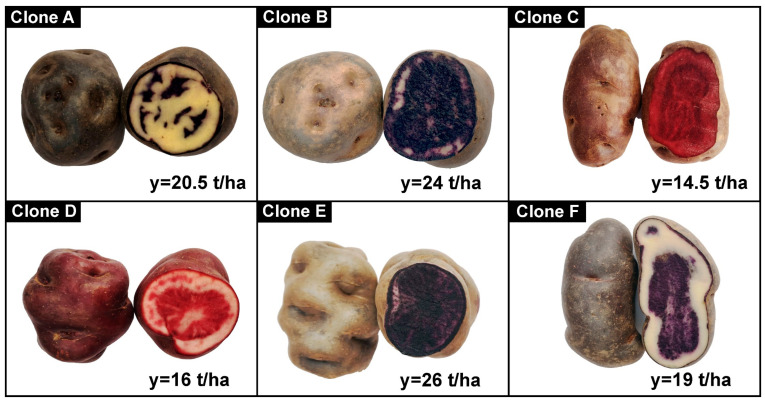
Native potato clones and yields (y).

Native potato clones selected according to weight and size were used in the first category (tubers between 91–120 g and 111–130 mm) and the second category (61–90 g and 91–110 mm); Figure 1 shows the pigments and yields of the native potato clones. All other reagents and inputs used in the laboratory met the quality requirements for use in the research protocols.

### 2.2. Obtaining Chips from Native Potato Clones

**Figure 2:**
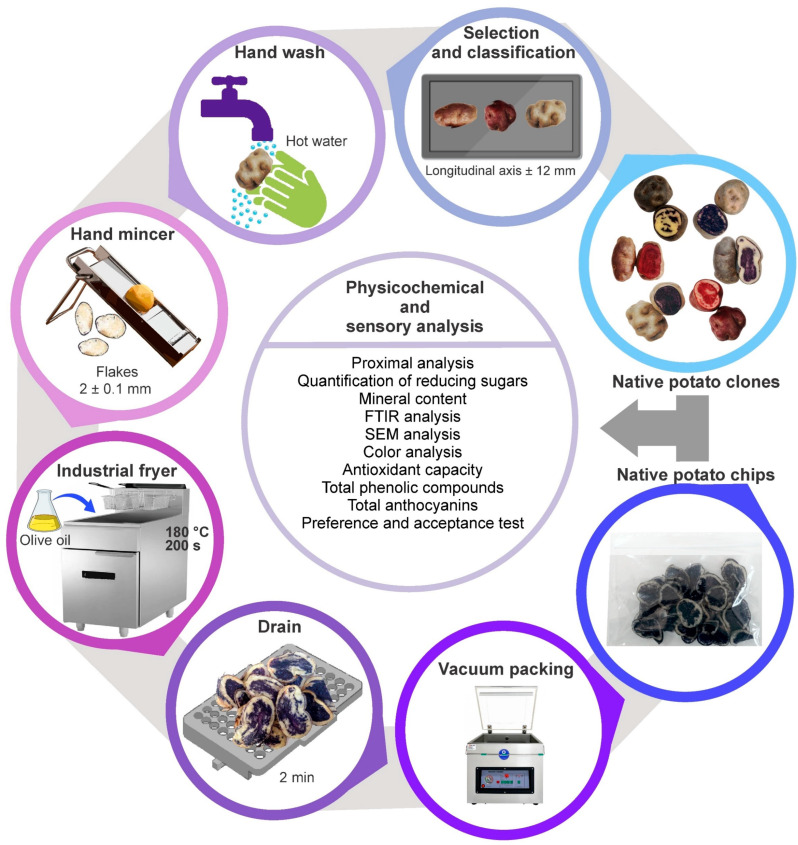
The process diagram for chip production.

First, the raw materials in lousy condition were separated. Then, the suitable native potato clones were selected and classified according to their size (longitudinal axis ± 12 mm), followed by manual washing with hot water to eliminate impurity residues. Subsequently, chips with an average thickness of 2 ± 0.1 mm were obtained using a manual grinder; the chips were fried by immersion in extra virgin olive oil packaged in high-density polypropylene of 2 L (brand Tottus, Lima, Perú) at a temperature of 180 °C and time of 200 s in an industrial fryer (model AEF-4050-S-E, Asber, Mexico) until homogeneous cooking was achieved, and then the oil was drained for 2 min in stainless steel strainers. Finally, the chips were cooled on absorbent paper to eliminate excess fat for 5 min and then packed in polypropylene bags, avoiding voiding excess air and sealing them hermetically. The chips obtained were stored until they were used in the physicochemical and sensory evaluation. Figure 2 shows the process diagram for getting chips.

### 2.3. Proximate Analysis

It was determined, according to AOAC standard methods (2012), measurements for moisture (AOAC 925.10), protein (AOAC 2003.05), fat (AOAC, 923.03), fiber (AOAC 985.29), and ash (AOAC 960.52) [22]. Carbohydrates were determined by difference.

### 2.4. Reducing Sugars

For the quantification of reducing sugars, Miller’s methodology was used. The DNS (3,5-dinitro salicylic acid) reagent was prepared by dissolving 11 g of NaOH, 10 g of DNS, 2 g of phenol, and 0.5 g of bisulfite in 100 mL of ultrapure water. Additionally, Rochelle’s salt was prepared with 40% sodium potassium tartrate. Amounts of 0.5 mL of sample, 3 mL of DNS, 1 mL of Rochelle salt, and 10 mL of distilled water were heated in a water bath at 100 °C, and the solution was homogenized with a vortex. The same procedure was performed for the blank using ultrapure water. Fructose was used for the calibration curve, and readings were performed in a spectrophotometer (Thermo Fisher Scientific, Waltham, MA, USA) at 550 nm. The results were expressed as mg/100 g dry basis [23,24].

### 2.5. Mineral Content

For microwave-assisted digestion, 0.5 g of each native potato clone, 3 mL of HCl, and 9 mL of HNO_3_ were taken, completing the volume to 50 mL with ultrapure water; digestion was performed at 180 °C for 10 min in a MiniWave microwave digester (SCP Science, Quebec, Canada). Samples were analyzed in an inductively coupled plasma atomic emission spectrophotometer ICP-OES 9820 138 (Shimadzu, Kyoto, Japan), with a sample exposure of 30 s and with an argon flow rate of 10 L/min in axial mode. The mineral content was expressed in mg/100 g of sample [19].

### 2.6. Color Analysis

The colorimetric values of the samples were determined by the reflectance module of the CR-5 colorimeter (Konica Minolta, Tokyo, Japan) using a Petri dish with a diameter of 30 mm. The results were expressed as the color parameters L*, a*, and b*. The color variation was calculated with the following formula.

(1) $$\Delta E_{ab}^{*}=\sqrt{\Delta L^{*2}+\Delta a^{*2}+\Delta b^{*2}}\;$$

where: $\Delta E_{ab}^{*}$ is the color variation, and $\Delta L^{*}$, $\Delta a^{*}$, and $\Delta L^{*}$ are the differences between L* a* b* of the reference and L* a* b* of the comparison [25,26,27].

### 2.7. Analysis by Fourier Transform Infrared Spectroscopy (FTIR)

IR spectra of native potato clones were obtained through the Nicolet IS50 FTIR transmission module (ThermoFisher, Waltham, MA, USA), using 2 mg of sample and 200 mg of KBr for pellet preparation. Readings were performed in the spectral range from 4000 to 400 cm^−1^ with the KBr beam splitter at a resolution of 8 cm^−1^ and with 32 scans.

For the case of the IR, spectra of native potato clone chips were obtained through the ATR-attenuated total reflectance module; the readings were performed in the mid-IR range at a resolution of 8 cm^−1^ and with 32 scans, using the advanced ATR correction for the diamond crystal, with an incidence angle of 45 and a refractive index of 1.5 [28].

### 2.8. Analysis by Scanning Electron Microscopy (SEM)

Microphotographs were obtained using a Prisma E scanning electron microscope (Thermo Fisher Scientific, Brno, Czech Republic). Carbon adhesive disks and 12.7 × 8 mm aluminum sample holders were used for sample preparation; the micrographs were observed under low vacuum at 0.07 Torr and a magnification of 100^x^ [26].

### 2.9. Antioxidant Capacity

The Trolox reagent (6-hydroxy-2,5,7,8-tetramethylchroman-2-carboxylic acid) was used for the calibration curve of antioxidant capacity by the DPPH (2,2-Diphenyl-1-Picrylhydrazyl) method. Methanolic extracts were prepared with 2 g of sample and 20 mL of 80% methanol, and they were left in the dark for 24 h at room temperature. After that, the DPPH solution was adjusted to an absorbance of 1.1 ± 0.02 read at a wavelength of 515 nm, and the UV spectrophotometer was brought to zero with methanol. For quantification, 150 µL of the sample extract was taken and allowed to react with 2850 µL of dilute DPPH solution for 15 min at room temperature. A blank was prepared using 150 µL of 80% methanol and 2850 µL of dilute DPPH solution. Readings were taken in quartz cuvettes at 515 nm, and the results were expressed on a dry basis of µmol ET/g sample [19,25,27,29].

### 2.10. Total Phenolic Compounds

Gallic acid was used for the calibration curve of phenolic compounds according to the Folin-Ciocalteu methodology. Methanolic extracts were prepared with 2 g of sample and 20 mL of 80% methanol, and they were left in the dark for 24 h at room temperature. A total of 3300 µL of methanolic extract, 150 µL of 20% Na_2_CO_3_, and 300 µL of 0.25 N Folin-Ciocalteu reagent were left to react for 15 min under dark conditions at room temperature; a blank was prepared under the same conditions using distilled water instead of the extract. Spectrophotometric readings were performed at 755 nm (Genesys 150, Thermo Fisher Scientific, Waltham, MA, USA), and the results were expressed on a dry basis as mg gallic acid equivalent (GAE)/g of sample [19,25,27,29].

### 2.11. Total Anthocyanins

The differential pH method of Giusti and Wrolstad was used to quantify total anthocyanins. Ethanolic extracts were prepared using 20 mL of extracting solvent (95% ethanol and 1% HCl) and 1 g of sample and allowed to react for 24 h. The samples were treated with 0.025 M KCl and 0.4 M C_2_H_3_NaO_2_ buffers, adjusting the pH to 1 and 4.5, respectively. Readings were performed at the maximum wavelength and 700 nm (Genesys 150, Thermo Fisher Scientific, Waltham, MA, USA), considering the dilution factor previously calculated with the KCl buffer. The results were expressed on a dry basis as mg Cyanidin-3-Glucoside (C3G)/g sample [19,29].

### 2.12. Preference and Acceptance Test

The sensory evaluation was carried out after four weeks of product storage, at times far from mealtimes. Portions of 10 g of the chips, identified with random three-digit numbers on the evaluation cards, were served to 90 panelists. The environment was well lit, without unpleasant odors, and with good ventilation. The untrained panelists (60% men and 40% women) were chosen between 21 and 42 years of age, since this is the age range that consumes more snacks, and they were also adults who are able to sign the ethical consent form [30].

A preference test was conducted in which consumers were asked which coded samples they preferred, even if unsure. The same 90 untrained panelists were given a product acceptability test, in which characteristics, such as color, smell, taste, and texture, were evaluated, using a 5-point hedonic scale with the following descriptors (I would not say I like = 1, I slightly dislike = 2, I neither like nor dislike = 3, I slightly like = 4, and I strongly like = 5) [18].

### 2.13. Statistical Analysis

To analyze the physicochemical data, analysis of variance and Fisher’s multiple range test at 5% significance were used; all results were obtained in triplicate. In the case of sensory evaluation, the Kolmogórov-Smirnov normality test was performed first, followed by the Kruskal-Wallis test. Origin Pro 2023 software (OriginLab Corporation, Northampton, MA, USA) was used for all statistical tests and the graphical representations.

## 3. Results and Discussion

### 3.1. Proximate Analysis

Table 1 shows the proximal composition of all fresh native potato clones, showing that moisture and carbohydrates showed significant differences between each clone (*p* ≤ 0.05); in the case of protein, fat, and fiber content, no significant differences were found (*p* > 0.05). Of particular interest is the dry matter content, since a higher value in this property and a lower level of reducing sugars contribute to the sensory characteristics and oil saving in frying [3,5], and the variation in the physicochemical properties studied is attributed to genotypic factors related to the variety, agroecological conditions, and crop tillage [31]. Regarding moisture content, similar values were obtained to those reported in native “*Puka Ambrosio*” potatoes by García et al. [4] and lower values than those reported in native pigmented potatoes from Chile (80–82.85%) [32] and potatoes destined for frying in China (80.48%) [15].

Clones B and A had the lowest moisture content; in the case of carbohydrate content, clones E and D had the lowest values; on the other hand, clones E and B have the lowest protein content. The properties above allowed us to have a previous idea of how the clones could behave during the technological development of the product, since a more significant amount of dry matter will allow an improvement in texture and more economical processes; in addition, a lower level of carbohydrates and proteins would allow a decrease in the appearance of melanoidins and acrylamide due to the effect of high temperatures [4,33]. Furthermore, the nutritional value of native potato clones could be highlighted on account of their content of fiber, essential amino acids, vitamins, minerals, and compounds with antioxidant potential, such as the ascorbic acid, α-tocopherol, carotenoids, different polyphenols, and phenolic acids [31,32]. Notorious comparative advantages were observed in native potato clones concerning commercial varieties, such as “*Blanca*,” “*Peruanita*”, “*Huayro*”, “*Huamantanga*”, and “*Canchan*”, which would demonstrate their suitability for the production of fried products [4].

Table 1 also shows the results of proximate composition in the chips; no significant differences were observed in the case of moisture (*p* > 0.05); the contrary was the case for the other properties studied (*p* ≤ 0.05). The dry matter contents were lower than those reported in native potato “*Puka Ambrosio*”; however, the carbohydrate and protein contents were similar to those of the clones studied [4].

Of particular interest was the fat content in which significant differences were observed between each clone (*p* ≤ 0.05), noting that clone B reported the lowest content (29.38%), followed by clone A with 31.36%; these values were higher than those reported in the native potato “*Puka Ambrosio”* by García et al. [4]. Previous studies have found that the decrease in oil absorption is conditioned by frying parameters, dry matter content, physicochemical properties, microstructure roughness, and pore size in potato slices used to obtain chips [15,34]. The low oil absorption would help prolong the product’s shelf life, reducing lipid oxidation that produces unpleasant flavors [5]. On the other hand, clones C and E are the ones that presented lower carbohydrate content and higher fiber content, which contribute to the nutritional benefits of these chips, despite their high-fat content.

### 3.2. Reducing Sugars

**Figure 3:**
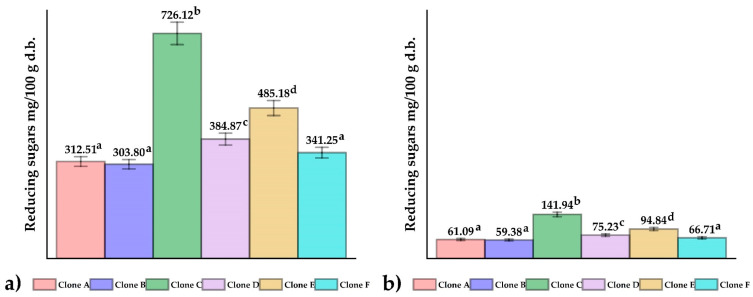
The reducing sugars content in (**a**) native potato clones and (**b**) chips. Different letters indicate significant differences.

Figure 3 shows the results of reducing sugars in fresh native potato clones (Figure 3a) and chips (Figure 3b); no significant differences were observed between samples A, B, and F (*p* > 0.05), whereas the opposite happened to clones C, D, and E (*p* ≤ 0.05). The values obtained in fresh were within the range reported for commercial potatoes intended for chip production (222.2–585.2 mg/100 g in dry basis) [35]. Reducing sugars are precursors of acrylamide, and their content is influenced by the environmental conditions of cultivation and the genetics of potatoes [36,37,38]; tubers intended for chip production should be stored between 8 and 12 °C, since values below this range increase the content of reducing sugars due to starch degradation [39]. The content of reduced sugars in French fries decreases by about 80%, concerning fresh potatoes, as a result of the interaction of temperature and time during frying (180 °C and 200 s), which would be related to a decrease in the amino acid asparagine and an increase in acrylamide [35].

### 3.3. Mineral Content

Table 2 shows the mineral content in fresh native potato clones and chips, showing that potassium was the highest, followed by magnesium and calcium; the other minerals found were reported at trace levels and even not detected. Regarding minerals in fresh native ecotypes, Villacres et al. [7] reported potassium and iron contents of 1741 mg/100 g and 6 mg/100 g, respectively; these values were higher than those reported in the present study. On the other hand, it was observed that the frying process increased mineral content, a product of water evaporation, and the incorporation of oil in the chips [40]. Iron and zinc are organic mineral cofactors of enzymes, such as catalase and superoxide dismutase, that have, as substrates, reactive oxygen species (ROS); on the other hand, selenium is of great importance in human biology as a cofactor of the enzymes glutathione peroxidase and thioredoxin reductase; its deficiency contributes to the appearance of degenerative diseases and psychiatric pathologies [20]. Iron, zinc, calcium, potassium, phosphorus, magnesium, and sodium levels were also reported in Peruvian potatoes used to obtain “*chuño*” [41]. Compared to other cereals and legumes, native potatoes show mineral contents that allow them to be considered as alternative sources of micronutrients related to antioxidant defense [20].

### 3.4. Color Analysis

Table 3 shows the color parameters in native potato clones and chips; significant differences were observed for L*, a*, and b* (*p* ≤ 0.05). Luminosity values were quite variable in potato clones and ranged between 21.13–67.48, similar to chips between 19.53–57.58, and previous works considered acceptable chips’ luminosity values to be higher than 55 [5,33]. Nevertheless, this parameter would be more applicable to yellow potatoes, but this was not the case in non-conventional pigmented potatoes, such as the clones used in the present study; even so, the color parameters were quite similar to the native potatoes “*Huevo de Indio*”, “*Kitipsho*”, “*Azúcar Cantina*”, and “*Tinkuy*”, reported by Natividad et al. [5]. In addition, it is known that a correct combination of time and temperature during frying produces good physical attributes, such as color, appearance, texture, and flavor, as well as the preservation of unstable bioactive compounds, such as vitamin C and carotenoids [42].

On the other hand, appreciable changes in ΔE*ab values were observed, attributed to temperature and frying time and the appearance of non-enzymatic browning, resulting from the Maillard reaction. The color differences were observed in the chips, and they are confirmed by parameters a* and b*, which changed, which would be attributed to anthocyanins’ degradation [12].

### 3.5. FTIR Analysis

**Figure 4:**
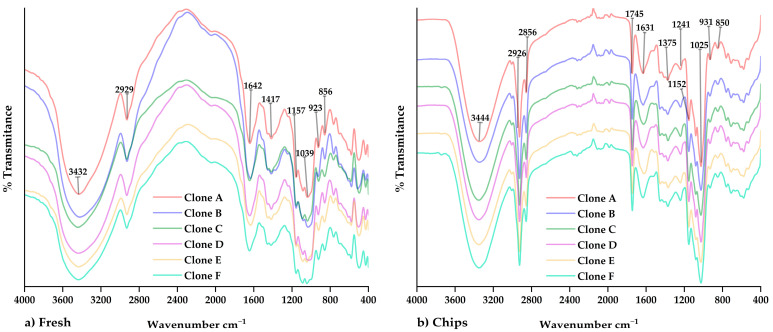
The FTIR spectra regarding (**a**) native potato clones and (**b**) chips of native potato clones.

Figure 4 shows the IR spectra of the native potato clones and chips, in which similar functional groups were observed in both fresh samples (Figure 4a) and processed samples (Figure 4b). The peak at 2856 cm^−1^ was observed in the chips, which would be attributed to the -CH_2_ and -CH_3_ groups in the fatty acid chains and their vibration by asymmetric stretching. In addition, the peak at 1745 cm^−1^ was observed, which would be related to the vibration of the carbonyl group and the formation of a complex between amylose and the lipids of the vegetable oil used during frying [15,43,44,45]. As well as that, the formation of a porous and cracked surface would favor the bonding of the amylose chain with the vegetable oil lipids, which can be observed by SEM [15].

### 3.6. SEM Analysis

**Figure 5:**
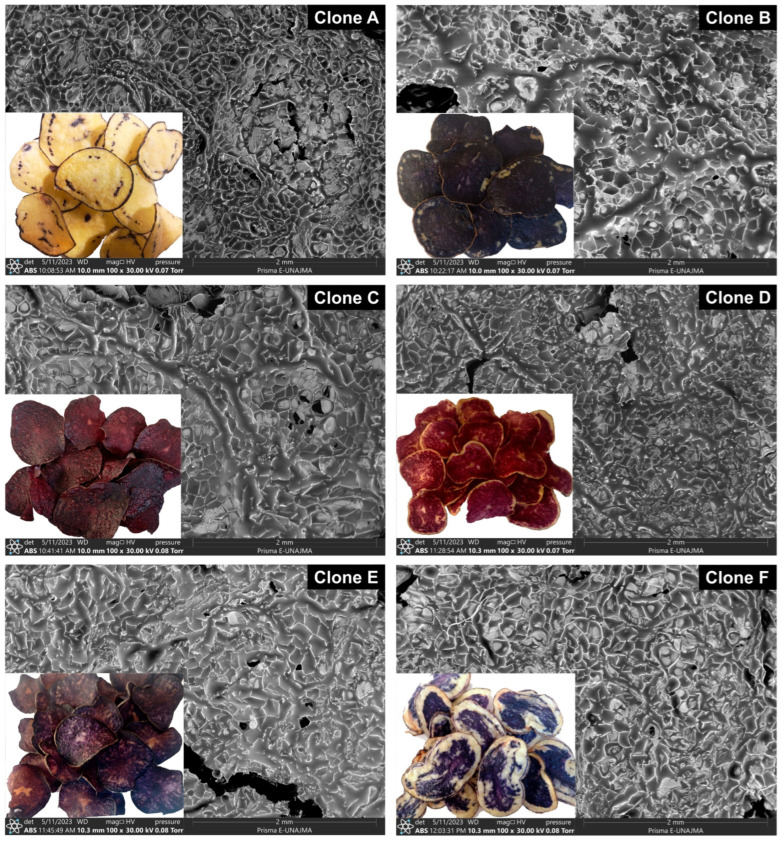
SEM micrographs of native potato clone chips.

Figure 5 shows the microphotographs of the chips of native potato clones at a magnification of 100^x^, in which the formation of microscopic pores and channels on the surface was observed, and the product of frying at a temperature of 180 °C and 200 s was observed, which is an indication that a good amount of water was removed by evaporation [15]; it is essential to study the rate of water evaporation during frying, since a rapid migration of steam causes further cracking of the surface and cell rupture [46].

As a result of the higher dry matter content in clones A and B, less cracking and cell rupture were observed in their chips, which would decrease the formation of the complex between amylose and frying oil lipids [10]. The topography of microphotographs in low-moisture products depends on the processing methods used; porous microstructures would indicate a higher dehydration rate, lower oil absorption, and a crispier chip texture [47,48]. Oil absorption is affected by the microstructure of the chip surface and the thickness of the slices used during frying [34]; in clones C and D, the appearance of melanoidins resulting from non-enzymatic browning is visibly observed [3].

On the other hand, the prolonged frying time of 200 s originated a change in the morphology of the chips, due in part to the gelatinization of the starch that created a rougher surface, combined with the use of a high temperature of 180 °C, which allowed the appearance of holes and cracks, which are quite visible in the SEM microphotographs [49].

### 3.7. Antioxidant Capacity, Phenolic Compounds, and Anthocyanins

Table 4 shows the results of bioactive compounds and antioxidant capacity, showing that clones B and C presented the highest values, both fresh and processed, in addition to an increase in the level of phenolic compounds and antioxidant capacity, which could be attributed to the degradation of anthocyanins that lead to the formation of various polyphenolic compounds, and hydrolysis of different compounds would also occur, including proteins, thus releasing phenolic compounds and making them more available, and, for this reason, it is recommended to study the interaction between anthocyanins and ascorbic acid during the frying operation of native potatoes [4].

### 3.8. Preference and Acceptance Test

**Figure 6:**
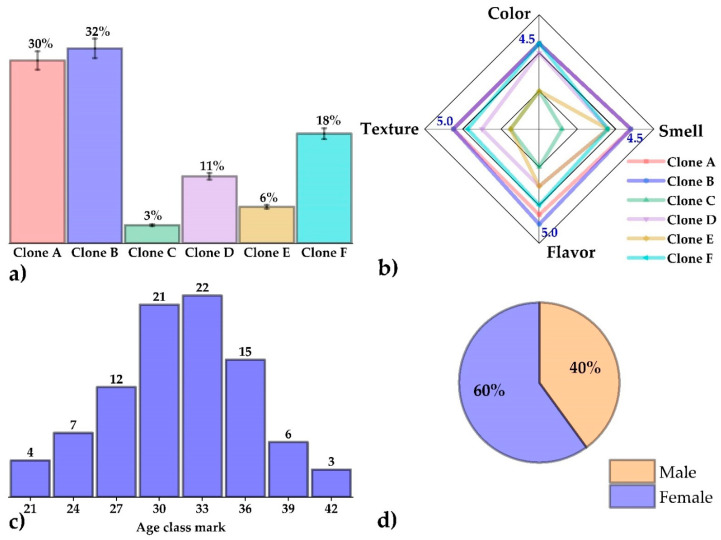
(**a**) Percentages of the preference test, (**b**) radar plot of the acceptance test, (**c**) histogram according to the age of the panelists, and (**d**) gender distribution of the panelists.

The results of the preference test are shown in Figure 6a, evidencing that clone B chips had the highest degree of preference (32%), followed closely by clone A (30%); it was also noted that clone C (3%) was the least preferred by the ninety untrained panelists consulted. In the acceptance test, the Kolmogorov-Smirnov normality test showed that all the results did not follow a normal distribution (*p* ≤ 0.05); also, the Kruskal-Wallis test evidenced statistical differences (*p* ≤ 0.05), which are shown in Table 5. The most important thing to note is that, for the attributes of color, odor, flavor, and texture, no significant differences (*p* > 0.05) were observed between the chips of clones A and B, both being mostly rated with the descriptor “I like it very much”, for all attributes. It is known that texture, aroma, color, and flavor are the leading quality indicators that define consumer preference and acceptance of this type of product [50].

Figure 6b shows that clones A and B presented the best results per descriptor and, therefore, were the most acceptable in the hedonic scale acceptance test; the ages of the ninety panelists were in a range between 21 and 42 years (Figure 6c), with 60% of the panelists being female and 40% male during the sensory evaluation (Figure 6d). It is known that ethnic or exotic flavors will play an essential role in food development in the future as more and more people travel the world and try new products, often influenced by economic, ethical, religious, and ancestral factors [18]. This attractiveness to consumers is primarily due to palatability, a set of gustatory, olfactory, and sensory experiences [19]. The product developed in the present study could be valued for its taste because it is derived from native Peruvian raw materials, which are increasingly present in the world for their nutritional and functional benefits.

### 3.9. Overview of Results on Native Potato Clone Chips

**Figure 7:**
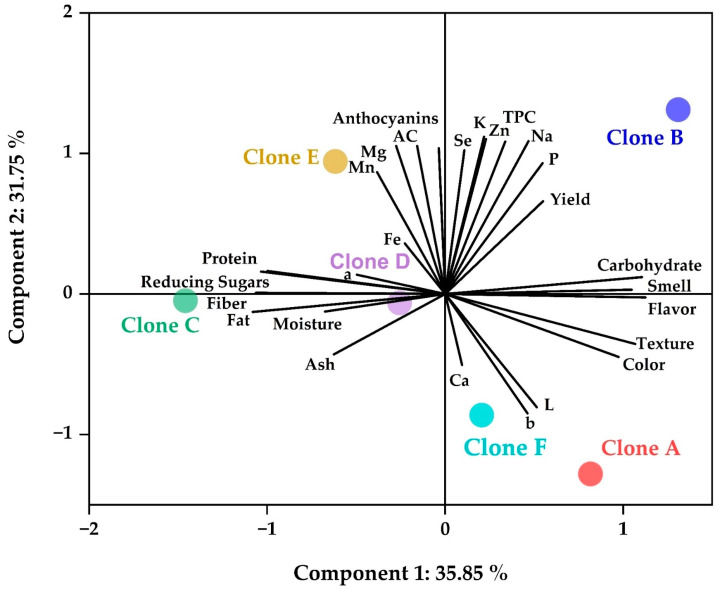
PCA study.

A principal component analysis (PCA) was performed on the results of the chips in order to observe the relationship between the complex variables [19,28]. Figure 7 shows that carbohydrates and sensory attributes are preferentially associated with clones A and B; on the other hand, the parameters of color L, b, and calcium are more related to clone F, and clones C, D, and E are associated with the properties studied in the proximal analysis and reducing sugars. Finally, clone B is more related to the remaining minerals, antioxidant capacity, bioactive compounds, and field performance.

## 4. Conclusions

Native potato clones grown in the high Andean region of Peru are a source of macronutrients, bioactive compounds, antioxidant capacity, and minerals; they have a high dry matter content and reduced levels of reducing sugars. When they are subjected to frying, their bioactive properties improve, increasing the levels of phenolic compounds and antioxidant capacity; on the other hand, the anthocyanin content is reduced. The formation of a smaller porous and cracked surface in the chips decreases the amylose binding with lipids, which was corroborated by FTIR and SEM analysis; correctly applying time and temperature during cooking produces desirable colors and attributes.

Finally, it is concluded that the chips of native potato clones present acceptable physicochemical and sensory quality, and fresh potatoes are a source of nutrients and bioactives with high yields in the field. Clone B had better physicochemical properties and greater sensory acceptance, followed by clone A; both could be used as raw material by food companies dedicated to producing snacks for the benefit of high Andean farmers.

## Figures and Tables

**Table 1:** Proximate analysis on native potato clones and chips.

Property	Clone A		Clone B		Clone C		Clone D		Clone E		Clone F	
	$\bar{x}$	±SD	$\bar{x}$	±SD	$\bar{x}$	±SD	$\bar{x}$	±SD	$\bar{x}$	±SD	$\bar{x}$	±SD
Fresh												
Moisture (%)	68.00 ^a^	±0.26	65.78 ^f^	±0.25	72.10 ^c^	±0.20	73.70 ^d^	±0.18	76.60 ^e^	±0.16	69.80 ^b^	±0.19
Protein (%)	1.32 ^a^	±0.21	1.30 ^a^	±0.31	1.43 ^a^	±0.17	1.56 ^a^	±0.19	1.24 ^a^	±0.23	1.45 ^a^	±0.21
Fat (%)	0.36 ^a^	±0.32	0.41 ^a^	±0.14	0.44 ^a^	±0.19	0.39 ^a^	±0.22	0.28 ^a^	±0.18	0.44 ^a^	±0.30
Ash (%)	0.97 ^ab^	±0.19	0.84 ^ab^	±0.20	0.75 ^a^	±0.13	0.87 ^ab^	±0.17	0.79 ^a^	±0.16	1.10 ^b^	±0.18
Fiber (%)	0.66 ^a^	±0.24	0.58 ^a^	±0.13	0.71 ^a^	±0.21	0.62 ^a^	±0.18	0.74 ^a^	±0.21	0.75 ^a^	±0.22
Carbohydrates (%)	28.70 ^a^	±0.22	31.10 ^b^	±0.15	24.60 ^c^	±0.18	22.90 ^d^	±0.21	20.30 ^e^	±0.22	26.50 ^f^	±0.19
Chips												
Moisture (%)	1.28 ^a^	±0.35	0.96 ^a^	±0.14	1.30 ^a^	±0.25	1.10 ^a^	±0.25	1.35 ^a^	±0.19	0.98 ^a^	±0.23
Protein (%)	2.38 ^a^	±0.28	2.09 ^a^	±0.19	3.36 ^b^	±0.18	3.45 ^b^	±0.18	3.36 ^b^	±0.21	2.42 ^a^	±0.15
Fat (%)	31.36 ^a^	±0.21	29.38 ^b^	±0.28	50.81 ^c^	±0.23	39.06 ^d^	±0.26	40.00 ^e^	±0.23	40.63 ^f^	±0.35
Ash (%)	1.74 ^ab^	±0.32	1.36 ^b^	±0.23	1.82 ^a^	±0.30	1.92 ^a^	±0.15	2.14 ^a^	±0.26	2.17 ^a^	±0.13
Fiber (%)	1.21 ^ac^	±0.19	0.93 ^a^	±0.17	1.66 ^b^	±0.17	1.41 ^cb^	±0.31	1.71 ^b^	±0.14	1.28 ^ac^	±0.25
Carbohydrates (%)	62.03 ^a^	±0.25	65.28 ^b^	±0.26	41.05 ^c^	±0.29	53.06 ^a^	±0.16	51.44 ^cd^	±0.13	52.52 ^e^	±0.18

Where: $\bar{x}$, arithmetic mean; SD, standard deviation. Different letters per row indicate significant differences, for *n* = 3.

**Table 2:** The mineral contents in native potato clones and chips.

Mineral (mg/100 g db)	Wavelength (nm)	Clone A		Clone B		Clone C		Clone D		Clone E		Clone F	
		$\bar{x}$	±SD	$\bar{x}$	±SD	$\bar{x}$	±SD	$\bar{x}$	±SD	$\bar{x}$	±SD	$\bar{x}$	±SD
Fresh													
K	767	635 ^a^	±3.06	1079 ^b^	±2.66	605 ^a^	±2.25	864 ^c^	±3.03	1069 ^b^	±2.35	455 ^a^	±2.07
Mg	280	32.06 ^a^	±2.68	53.52 ^b^	±2.44	47.02 ^b^	±2.24	35.36 ^a^	±3.11	71.46 ^c^	±5.78	27.99 ^a^	±1.22
Ca	185	13.70 ^a^	±0.20	5.53 ^b^	±0.06	7.83 ^c^	±0.12	8.20 ^d^	±0.10	6.30 ^e^	±0.01	2.53 ^f^	±0.06
P	252	0.86 ^a^	±0.03	3.84 ^b^	±0.02	0.09 ^c^	±0.03	2.87 ^d^	±0.06	2.30 ^e^	±0.06	0.54 ^f^	±0.03
Mn	258	0.28 ^a^	±0.01	0.35 ^b^	±0.01	0.35 ^b^	±0.01	0.39 ^c^	±0.02	0.47 ^d^	±0.01	0.15 ^e^	±0.01
Na	589	ND		2.68 ^a^	±0.01	0.54 ^b^	±0.01	1.02 ^c^	±0.03	1.02 ^c^	±0.01	0.43 ^d^	±0.03
Se	196	ND		0.17 ^a^	±0.12	ND		ND		0.27 ^a^	±0.06	ND	
Fe	240	0.03 ^a^	±0.01	0.07 ^b^	±0.01	0.06 ^c^	±0.01	0.17 ^d^	±0.02	0.07 ^e^	±0.02	0.04 ^f^	±0.01
Zn	214	ND		0.22 ^a^	±0.01	ND		0.05 ^b^	±0.01	0.26 ^a^	±0.03	ND	
Chips													
K	767	1440 ^a^	±2.28	1961 ^ab^	±4.23	1960 ^ab^	±1.95	2540 ^b^	±1.98	2384 ^b^	±2.49	1485 ^a^	±2.76
Mg	280	59.02 ^a^	±0.01	100 ^b^	±0.26	103 ^b^	±0.19	82.10 ^ab^	±0.19	74.01 ^ab^	±0.06	83.10 ^ab^	±0.19
Ca	185	50.15 ^a^	±0.15	107 ^b^	±0.58	65.02 ^a^	±0.17	50.11 ^a^	±0.20	58.31 ^a^	±0.17	83.57 ^a^	±0.46
P	252	2.01 ^a^	±0.01	4.21 ^b^	±0.01	1.15 ^a^	±0.01	3.05 ^b^	±0.01	3.14 ^b^	±0.01	1.03 ^a^	±0.01
Mn	258	4.04 ^b^	±0.01	1.21 ^a^	±0.01	0.81 ^a^	±0.01	0.61 ^a^	±0.01	0.62 ^a^	±0.01	0.81 ^a^	±0.01
Na	589	46.18 ^a^	±0.19	139 ^b^	±0.82	51.11 ^a^	±0.04	41.12 ^a^	±0.02	45.05 ^a^	±0.04	41.10 ^a^	±0.15
Se	196	ND		0.31 ^a^	±0.01	ND		ND		0.33 ^a^	±0.01	ND	
Fe	240	2.12 ^a^	±0.01	3.08 ^a^	±0.02	3.06 ^a^	±0.02	2.10 ^a^	±0.01	2.16 ^a^	±0.01	4.11 ^a^	±0.03
Zn	214	5.03 ^a^	±0.03	8.11 ^b^	±0.05	3.08 ^a^	±0.01	5.12 ^a^	±0.04	7.12 ^b^	±0.05	4.02 ^a^	±0.03

Where: $\bar{x}$, arithmetic mean; SD, standard deviation; ND, not detected; db, dry basis. Different letters per row indicate significant differences, for *n* = 3.

**Table 3:** The color results in native potato clones and chips.

Parameters	Clone A		Clone B		Clone C		Clone D		Clone E		Clone F	
	$\bar{x}$	±SD	$\bar{x}$	±SD	$\bar{x}$	±SD	$\bar{x}$	±SD	$\bar{x}$	±SD	$\bar{x}$	±SD
Fresh												
*L* *	67.48 ^a^	±0.06	21.91 ^b^	±0.15	26.63 ^c^	±0.25	36.65 ^d^	±0.46	21.13 ^e^	±0.31	24.24 ^f^	±0.58
*a* *	1.54 ^a^	±0.06	0.62 ^b^	±0.10	13.11 ^c^	±0.24	14.31 ^d^	±0.26	2.37 ^e^	±0.19	2.98 ^f^	±0.58
*b* *	36.16 ^a^	±0.16	−4.35 ^b^	±0.03	3.62 ^c^	±0.15	4.94 ^d^	±0.07	−3.16 ^e^	±0.12	−3.92 ^f^	±0.28
Chips												
*L* *	57.58 ^a^	±0.21	24.43 ^b^	±0.66	19.53 ^c^	±0.07	31.06 ^d^	±0.27	20.70 ^e^	±0.12	40.09 ^f^	±0.11
*a* *	0.25 ^a^	±0.21	0.82 ^b^	±0.13	4.95 ^c^	±0.10	15.73 ^d^	±0.13	4.79 ^c^	±0.10	1.00 ^b^	±0.08
*b* *	25.12 ^a^	±0.26	1.11 ^b^	±0.68	0.26 ^c^	±0.01	6.99 ^d^	±0.13	1.82 ^e^	±0.08	2.86 ^f^	±0.27
ΔE*_ab_	14.86 ^a^	±0.15	6.02 ^b^	0.80	11.33 ^c^	0.26	6.12 ^b^	0.16	5.53 ^b^	0.10	17.36 ^d^	0.38

Where: $\bar{x}$, arithmetic mean; SD, standard deviation. Different letters per row indicate significant differences, for *n* = 3.

**Table 4:** Antioxidant capacity, phenolic compounds, and anthocyanins in native potato clones and chips.

Clones	Clone A		Clone B		Clone C		Clone D		Clone E		Clone F	
	$\bar{x}$	±SD	$\bar{x}$	±SD	$\bar{x}$	±SD	$\bar{x}$	±SD	$\bar{x}$	±SD	$\bar{x}$	±SD
Fresh												
AC µmol ET/g db	867 ^a^	±2.28	929 ^b^	±2.83	902 ^b^	±1.78	879 ^c^	±2.48	887 ^d^	±2.51	857 ^a^	±8.27
TPC mg GAE/g db	20.37 ^a^	±1.91	27.38 ^b^	±2.56	23.19 ^a^	±2.23	22.00 ^a^	±1.74	22.82 ^a^	±2.14	21.44 ^a^	±0.08
Antocyanins mg C3G/g db	0.43 ^a^	±0.02	2.60 ^b^	±0.01	1.98 ^c^	±0.02	1.43 ^d^	±0.03	1.58 ^e^	±0.04	1.44 ^d^	±0.02
Chips												
AC µmol ET/g db	884 ^a^	±2.37	938 ^b^	±3.74	929 ^b^	±2.74	897 ^c^	±3.41	914 ^d^	±1.51	883 ^a^	±8.27
TPC mg GAE/g db	28.93 ^a^	±2.62	39.15 ^b^	±3.04	32.70 ^a^	±2.08	31.46 ^a^	±2.30	32.40 ^a^	±1.88	30.01 ^a^	±0.08
Antocyanins mg C3G/g db	0.20 ^a^	±0.01	1.17 ^b^	±0.02	0.87 ^c^	±0.03	0.67 ^d^	±0.02	0.71 ^e^	±0.01	0.65 ^d^	±0.02

Where: $\bar{x}$, arithmetic mean; SD, standard deviation; db, dry basis. Different letters per row indicate significant differences, for *n* = 3.

**Table 5:** Scoring by attributes in the sensory acceptance test of chips.

Attribute	Clone A		Clone B		Clone C		Clone D		Clone E		Clone F	
	Descriptor	$\tilde{x}$	Descriptor	$\tilde{x}$	Descriptor	$\tilde{x}$	Descriptor	$\tilde{x}$	Descriptor	$\tilde{x}$	Descriptor	$\tilde{x}$
Color	I like it very much	4.5 ^a^	I like it very much	4.5 ^a^	I dislike it slightly	2.0 ^b^	I like it slightly	4.0 ^c^	I dislike it slightly	2.0 ^b^	I like it very much	4.5 ^a^
Smell	I like it very much	4.5 ^a^	I like it very much	4.5 ^a^	Neither like nor dislike	3.0 ^b^	I like it slightly	4.0 ^c^	I like it slightly	4.0 ^c^	I dislike it slightly	4.0 ^c^
Flavor	I like it very much	4.5 ^a^	I like it very much	5.0 ^a^	I dislike it slightly	2.0 ^b^	I like it slightly	3.0 ^c^	Neither like nor dislike	3.0 ^c^	I like it very much	4.0 ^d^
Texture	I like it very much	5.0 ^a^	I like it very much	5.0 ^a^	Neither like nor dislike	3.0 ^b^	I like it slightly	4.0 ^c^	Neither like nor dislike	3.0 ^b^	I like it very much	4.5 ^a^

Where: $\tilde{x}$, median. Different letters per row indicate significant differences.

## Data Availability

Data is contained within the article.
